# Influence of Magnolol on the Secretion of α-Toxin by *Staphylococcus aureus*

**DOI:** 10.3390/molecules15031679

**Published:** 2010-03-12

**Authors:** Hua Xiang, Jia-Zhang Qiu, Da-Cheng Wang, You-Shuai Jiang, Li-Jie Xia, Xu-Ming Deng

**Affiliations:** 1 Institute of Zoonoses, College of Animal Sciences and Veterinary Medicine, Jilin University, Changchun, Jilin 130062, China; E-Mails: xianghuaxh@126.com (H.X.); qiujiazhang1983@163.com (J.-Z.Q.); 2 College of Life Science and Technology, Heilongjiang August First Agricultural University, Daqing, Heilongjiang 163319, China; E-Mail: jiangyoushuai10@yahoo.com.cn (Y.-S.J.); 3 College of Veterinary Medicine, Yangzhou University, Yangzhou, Jiangshu 225009, China; E-Mail: castle19850816@yahoo.com.cn (L.-J.X.)

**Keywords:** magnolol, *Staphylococcus aureus*, α-toxin

## Abstract

In this study we investigated the antimicrobial activity of magnolol on *Staphylococcus aureus*. The minimal inhibitory concentrations of magnolol against 31 *S. aureus* strains ranged from 4–32 μg/mL. In addition, hemolysin assays, Western blotting, and real-time RT-PCR were performed to investigate the effect of magnolol on α-toxin secretion by both methicillin-sensitive *S. aureus* (MSSA) and methicillin-resistant *S. aureus* (MRSA). The results indicated that sub-inhibitory concentrations of magnolol dose-dependently inhibited the transcription of *hla* (the gene encoding α-toxin) in *S. aureus*, resulting in a reduction of α-toxin secretion and, thus, hemolytic activities.

## 1. Introduction

*Staphylococcus aureus* is a leading cause of nosocomial infections and the pathogen responsible for a variety of diseases associated with significant morbidity and mortality. The diseases originating from this species include skin and soft tissue lesions, as well as lethal infections such as osteomyelitis, endocarditis, pneumonia, and septicemia [1]. The continuous emergence of methicillin-resistant *S. aureus* (MRSA), glycopeptide-insensitive *S. aureus* (GISA), and vancomycin-resistant *S. aureus* (VRSA) strains have made it difficult to treat *S. aureus* infections [2]. Therefore, there is an urgent need to develop and employ alternative antimicrobial agents or therapeutic strategies to control these life-threatening infections.

Like other Gram-positive bacteria, the pathogenicity of *S. aureus* is largely dependent upon extracellular virulence factors, including both secreted and surface proteins [3]. One of the most important extracellular proteins is α-toxin, a pore-forming, soluble 33-kDa protein that is secreted by most *S. aureus* strains, primarily during the post-exponential phase. Like most staphylococcal exotoxins, α-toxin is regulated by a number of extracellular and intracellular signals, including the SarA protein family [4] and numerous two-component regulatory systems, such as *agr* [5] and *sae* [6]. The toxin can cause pore formation of a wide range of human cells, including erythrocytes, monocytes, lymphocytes, macrophages, and epithelial cells. Furthermore, it can also induce pro-inflammatory changes in mammalian cells. The consequent cellular damage may contribute to manifestations of the sepsis syndrome.

**Figure 1:**
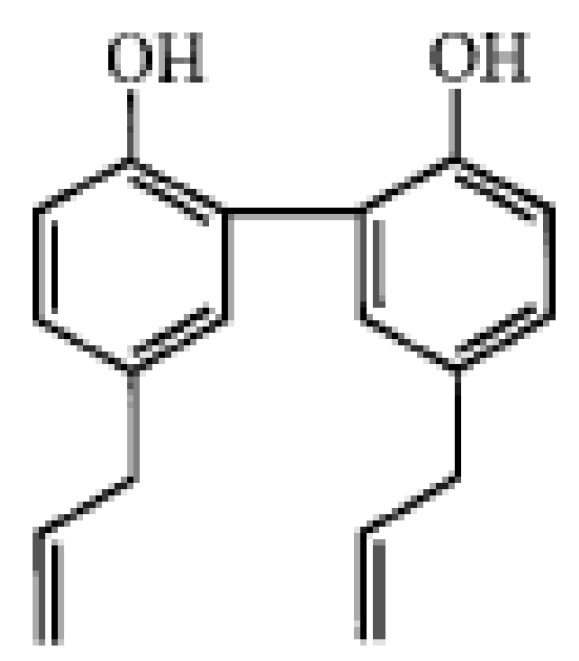
Structure of magnolol.

Magnolol (Figure 1), the main phenolic component of the Chinese medicinal herb *Magnolia officinalis*, has been shown to possess a variety of pharmacological activities, including anti-inflammatory, antimicrobial, anti-allergic, and anti-asthmatic activities [7,8,9]. The present study aimed to assess the antimicrobial activity of magnolol on *S. aureus* and to investigate the effect of sub-inhibitory concentrations of magnolol on α-toxin expression by both methicillin-sensitive and methicillin-resistant *S. aureus*.

## 2. Results and Disscussion

### 2.1. Influence of magnolol on S. aureus growth

**Figure 2:**
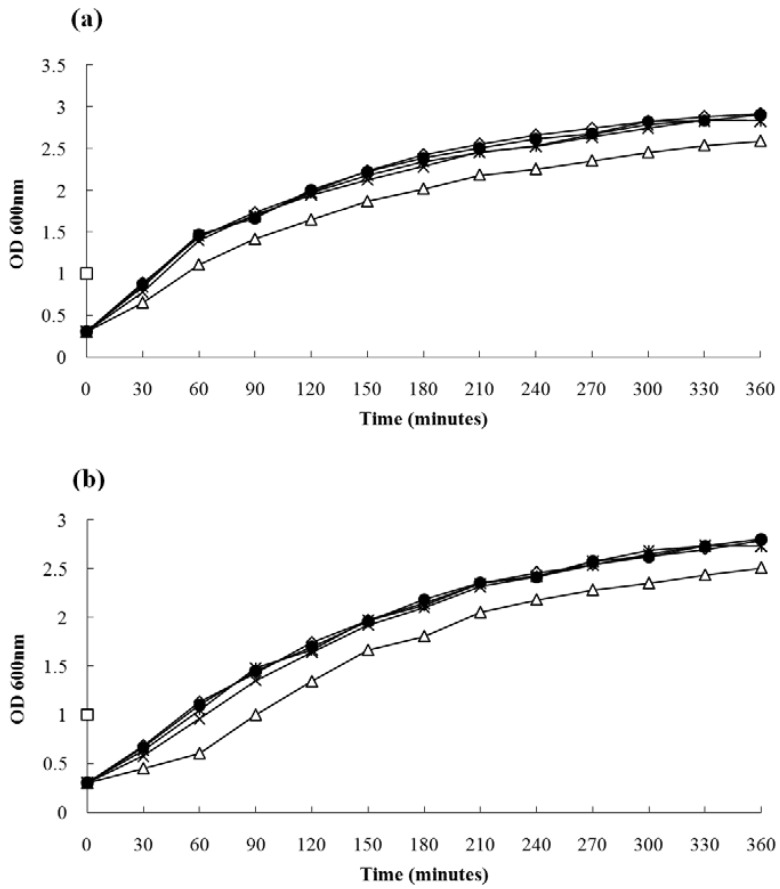
Growth curves for *S. aureus* strain ATCC 29213 (a) and MRSA 3701 (b) in the presence or absence of magnolol. ◇, ●, ×, _*_, and △— Strain ATCC 29213 and MRSA 3701 grown in LB plus 0, 1, 2, 4, and 8 μg/mL magnolol, respectively.

The MIC of magnolol for each of the 31 *S. aureus* strains ranged from 4–32 μg/mL, and the MIC values of *S. aureus* strain ATCC 29213 and MRSA strain 3701 *vs.* magnolol were 16 μg/mL. These data are in line with previous reports [7], suggesting that magnolol may be useful to treat *S. aureus* infections. The growth characteristics of *S. aureus* ATCC 29213 in the presence of magnolol are shown in Figure 2a, wherein we found that 1/16 × MIC, 1/8 × MIC, and 1/4 × MIC of magnolol had little influence on the growth of *S. aureus*, after 360 min of magnolol treatment, the OD_600 nm_ values were 99.5, 96.9, and 95.8% of the control culture, respectively. However, the growth velocity of *S. aureus* cultured with 1/2 × MIC of magnolol was significantly decreased; after 60, 180, and 360 min of magnolol treatment, the OD_600 nm_ values were 77.1, 83.1, and 88.6% of the control culture, respectively. The growth features of MRSA strain 3701 with different concentrations of magnolol were similar to MSSA ATCC 29213 (Figure 2b).

### 2.2. Magnolol reduces the level of α-toxin expression by S. aureus

**Figure 3:**
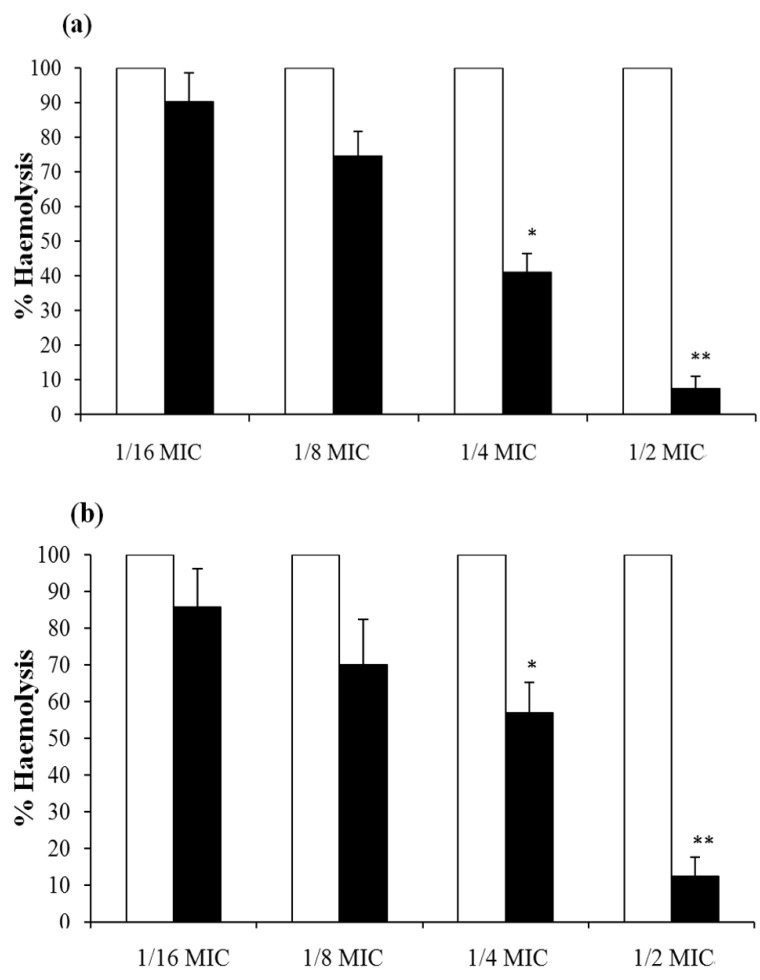
Haemolysis of rabbit erythrocytes by supernatants of cultures of MSSA strain ATCC 29213 (a) and MRSA strain 3701 (b) in the absence (□) or presence (■) of different concentrations of magnolol. Values represent the mean of three independent experiments, and the error bars show standard deviations. * represents *p *< 0.05, and ** represents *p *< 0.01.

The influence of magnolol on *S. aureus* α-toxin secretion was confirmed by phenotypic, expressional, and transcriptional analyses. Both strain ATCC 29213 and MRSA strain 3701 were grown in magnolol-free medium and in the presence of graded sub-optimal concentrations of magnolol. The culture supernatants were then used for investigating the hemolytic activities on rabbit erythrocytes. As shown in Figure 3, a dose-dependent inhibition of haemolysis was observed in both strains. The haemolytic activities of *S. aureus* 29213 when grown in the presence of 1/16 MIC, 1/8 MIC, 1/4 MIC, and 1/2 MIC of magnolol were 7.4, 40.8, 74.4, and 90.3% of the control culture, respectively. As for MRSA strain 3701, the respective values were 12.4, 57, 70, and 85.6%. Moreover, pre-incubation of culture supernatants with a 2 × MIC concentration of magnolol indicated that the compound had no obvious influence on hemolytic activities (data not shown). 

**Figure 4:**
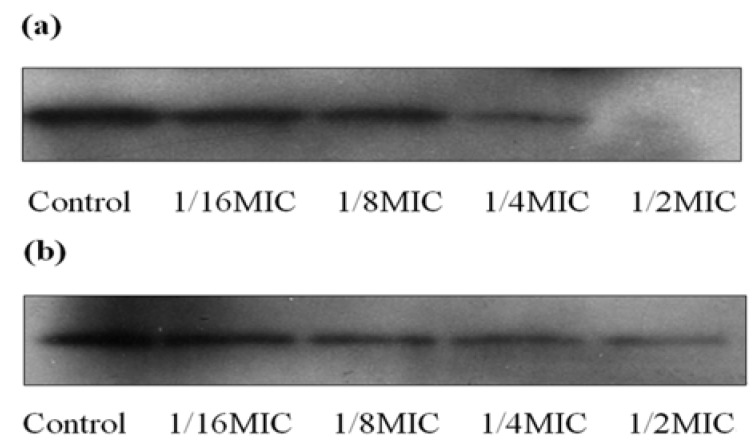
Western blot analysis of α-toxin secretion by strain ATCC 29213 (a) and MRSA strain 3701 (b) after growth with increasing concentrations of magnolol.

The effect of increasing concentrations of magnolol on the secretion of α-toxin by *S. aureus* was detected by Western blot analysis. Growth with 1/16 × MIC of magnolol resulted in a significant reduction in α-toxin by both strains; while at1/2 × MIC, no immunoreactive protein could be detected in strain ATCC 29123, and little of the protein could be found in MRSA strain 3701 (Figure 4). As expected, magnolol decreased the secretion of α-toxin in a dose-dependent manner. 

**Figure 5:**
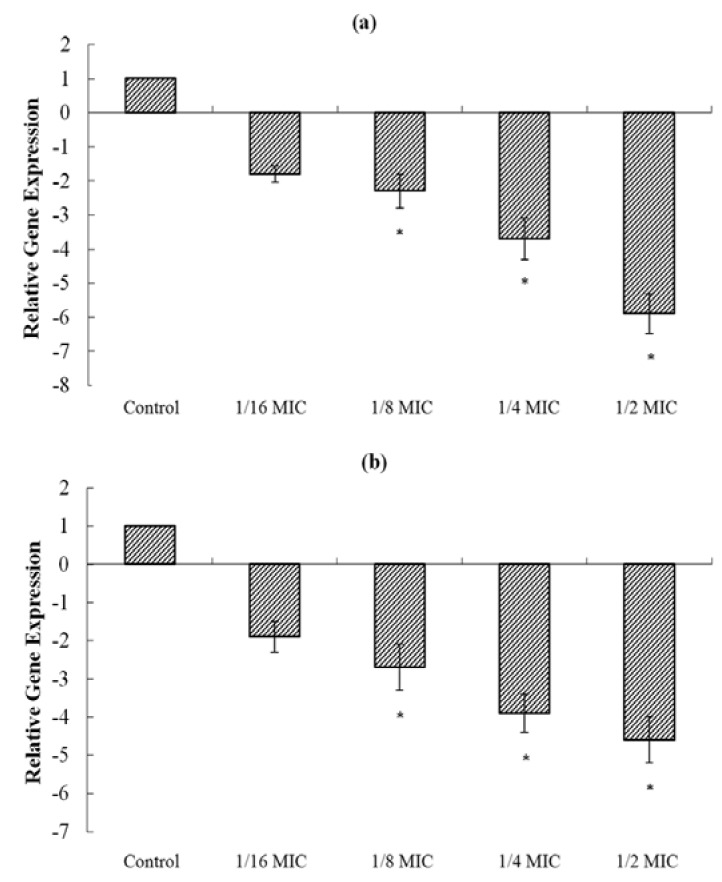
Relative gene expression of *hla* in strain ATCC 29213 (a) and MRSA strain 3701 (b) after growth with sub-inhibitory concentrations of magnolol. Values represent the mean and standard error of three independent experiments. * represents *p *< 0.05.

Real-time RT-PCR was carried out to assess the transcriptional level of the α-toxin encoding gene (*hla*) after treatment with sub-inhibitory concentrations of magnolol. As shown in Figure 5, magnolol significantly inhibited the transcription of *hla* in both *S. aureus* strains ATCC 29213 and MRSA 3701. 

When cultured with 1/16, 1/8, 1/4, and 1/2 × MIC of magnolol, the transcriptional levels of *hla* in strain ATCC 29213 were decreased by 1.8-, 2.3-, 3.7-, and 5.9-fold, respectively. As for MRSA strain 3701, the respective values were 1.9-, 2.7-, 3.9-, and 4.6-fold. The *hla* gene was affected by magnolol at the transcriptional level in a dose-dependent manner. 

### 2.3. Discussion

The oriental herb *M. officinalis* has been used in traditional Chinese and Japanese medicine for the treatment of acute pain, diarrhea, coughs, and urinary problems due to its wide-ranging therapeutic properties [8]. Magnolol, one of the bioactive constituents of *M. officinalis*, has attracted a number of research interests because of its pharmacological activities. In the study, we showed that magnolol was active against both MSSA and MRSA (with MICs ranging from 4–16 µg/mL), indicating that magnolol is a potentially effective antimicrobial agent against *S. aureus*. In addition, magnolol may be used as a lead compound for the design of more potent antibacterial agents to treat drug-resistant *S. aureus* strains.

The exoproteins of *S. aureus* can cause a number of diseases. For instance, α-toxin is capable of producing extensive corneal disease [10]; enterotoxins could result in staphylococcal gastoenteritis and are one cause of food poisoning in humans [11,12]; toxic shock syndrome toxin 1 (TSST-1) is the major causative toxin of toxic shock syndrome (TSS), which is a life-threatening staphylococcal infection [13]. Consequently, the clinical effects of antibiotics for the treatment of *S. aureus* are not only determined by their respective bacteriostatic or bactericidal activities but also by their abilities to prevent virulence factor release by dying or stressed bacteria. Many antibiotics, particularly when used at sub-optimal concentrations, may alter the secretion of virulence factors by *S. aureus*. For instance, linezolid, quinupristin/dalfopristin, and clindamycin significantly inhibit the production of virulence factors (including α-toxin, SEA, SEB, and protein A) in *S. aureus* at sub-growth inhibitory concentrations [3,14,15]. In contrast, some β-lactams and glycopeptide agents induce the expression of α-toxin, enterotoxins, and TSST-1 through a stimulatory effect on exoprotein synthesis [16,17], indicating that the symptoms of infections caused by *S. aureus* may be aggravated when patients are treated with these antibiotics. Some plant compounds (e.g., epicatechin gallate and licochalcone A) and essential oils (e.g., oils of bay, cinnamon, and clove) can also influence the production of exotoxins when used at sub-inhibitory concentrations [18,19,20]. In the present study, magnolol was shown to inhibit α-toxin expression by *S. aureus* via hemolysin assays, western blot analysis, and real-time RT-PCR. These data suggest that magnolol are potentially useful for the treatment of *S. aureus* infections when used in combination with β-lactams or glycopeptide antibiotics, which induce α-toxin expression in *S. aureus* at sub-inhibitory concentrations.

Targeting bacterial virulence is an alternative strategy that is now gaining great interest in antimicrobial therapy and offers promising opportunities to impede pathogenesis and its consequences without placing immediate life-or-death pressure on the target bacterium [21]. To date, many studies have shown that *S. aureus* has the ability to increase virulence, especially by MRSA, which may result in a more severe and widespread disease [22]. Furthermore, it has also been demonstrated that virulence factors can be transferred between *S. aureus* and *Staphylococcus epidermidis*, which may result in the transformation of *S. epidermidis* from a commensal bacterium into a more aggressive opportunistic pathogen [23]. These findings indicate an urgent need to develop antibiotics that target virulence factors. Our results showing that magnolol significantly decreases α-toxin secretion in *S. aureus* suggest that the structure of magnolol may be used as a basic structure for the development of drugs aimed at bacterial virulence factors.

## 3. Experimental

### 3.1. Bacterial strains and reagents

Thirty clinical *S. aureus* isolates (16 MSSA and 14 MRSA) were isolated at the First Hospital of Jilin University from blood samples of infected patients; the clinical MRSA strain 3701, which has the ability to secrete α-toxin, was chosen for further experiments. MSSA strain ATCC 29213 was obtained from the American Type Culture Collection (ATCC). Luria-Bertani (LB) broth was purchased from BD Biosciences, Inc. (Sparks, MD, USA). Magnolol was purchased from the National Institute for the Control of Pharmaceutical and Biological Products (Beijing, China), and stock solutions of various concentrations were made in dimethyl sulfoxide (DMSO) (Sigma-Aldrich, St. Louis, MO, USA).

### 3.2. Determination of Minimal Inhibitory Concentration (MIC)

The MICs of magnolol for *S. aureus* were determined in triplicate by a broth micro-dilution method, as recommended by the Clinical and Laboratory Standards Institute. The MICs were defined as the lowest drug concentration that inhibited growth.

### 3.3. Growth curves

*S. aureus* strain ATCC 29213 was cultured at 37 ºC to an OD value of 0.3 at 600 nm in LB, and 100-mL volumes of the culture were placed into five 250-mL Erlenmeyer flasks. Magnolol (dissolved in DMSO) was added to four of the cultures to obtain final concentrations of 1/16 × MIC (1 µg/mL), 1/8 × MIC (2 µg/mL), 1/4 × MIC (4 µg/mL), and 1/2 × MIC (8 µg/mL). The final DMSO concentration for all cultures was 1‰ (v/v). The control culture contained 1‰ DMSO alone. Bacteria were further grown at 37 ºC with constant shaking under aerobic conditions, and cell growth was monitored by reading the OD_600 nm_ values at 30-min intervals. 

### 3.4. Hemolysin assay

α-Toxin levels in bacterial culture supernatants were determined according to the method of Rowe and Welch [24]. Bacteria were cultured at 37 ºC with graded sub-inhibitory concentrations of magnolol to the post-exponential growth phase (OD_600 nm_ of 2.5, equivalent to 1 × 10^9^ CFUs/mL). Bacterial samples were centrifuged (5,500 × g, 4 ºC, 1 min), the supernatant was removed, and 0.1 mL culture supernatant was brought up to 1 mL in hemolysin buffer (0.145 mol/L NaCl, 0.02 mol/L CaCl_2_) prior to the addition of 25 µL of defibrinated rabbit blood. After incubation for 15 min at 37 ºC, the unlysed blood cells were pelleted by centrifugation (5,500 × g, room temperature, 1 min). The hemolytic activity of the supernatant was determined by measuring the optical densities at 543 nm. The control culture supernatant served as 100% haemolysis, and the % haemolysis was calculated by comparison with the control culture. 

### 3.5. Western blot assay

*S. aureus* strains were grown, and supernatant samples were prepared in the same manner as for the hemolysin assay. Samples (16 µL) of supernatant fluid were loaded on a 12% sodium dodecyl sulfate-polyacrylamide gel after boiling in Laemmli sample buffer [25]. For western blot analysis, proteins were transferred to polyvinylidene fluoride membranes (Wako Pure Chemical Industries, Ltd., Osaka, Japan) using a semi-dry transfer cell (Bio-Rad, Munich, Germany). Following blotting of the membranes, blocking was performed with 5% BSA (Wako) in phosphate-buffered saline for 2 h. The filters were then incubated for 1 h with a polyclonal anti-α-toxin antibody (Sigma-Aldrich) in phosphate-buffered saline containing 0.05% Tween-20, followed by 0.5 h of incubation with horseradish peroxidase-conjugated anti-rabbit antiserum (Sigma-Aldrich) diluted 1:4,000. The blots were developed using the ECL substrate (GE Healthcare, UK).

### 3.6. Real-time RT-PCR

Strain ATCC 29213 was grown in LB at 37 ºC with graded sub-inhibitory concentrations of magnolol to the post-exponential growth phase (t = 240 min). RNA was isolated as described by Sambanthamoorthy [26]. Briefly, cells were collected by centrifugation (5,000 × g for 5 min at 4 ºC) and resuspended in TES buffer (10 mM Tris-Cl, 1 mM EDTA, 0.5% SDS) including 100 µg/mL lysostaphin (Sigma-Aldrich). Following incubation at 37 ºC for 10 min, a Qiagen RNeasy Maxi column was used to isolate total bacterial RNA in accordance with the manufacturer’s directions. The optional on-column RNase-free DNase I (Qiagen, Hilden, Germany) treatment was carried out to remove contaminating DNA. After isolation of RNA, traces of contaminating DNA were further eliminated by treating RNA samples with RNase-free DNase I (Ambion, Austin, TX) at 37 ºC for 20 min. RNA concentrations were determined from the OD260 nm, and the RNA was run on an RNase-free 2% agarose gel to test for generalized degradation. The primer pairs used in real-time RT-PCR are listed in Table 1. RNA was reverse transcribed into cDNA using the Takara RNA PCR kit (AMV) Ver. 3.0 (Takara, Kyoto, Japan), according to the manufacturer’s instructions; cDNA was stored at -20 ºC until needed. The PCR reactions were performed in a 25-µL final volume and contained SYBR Premix Ex TaqTM (Takara), as recommended by the manufacturer. The reactions were carried out by using the 7000 Sequence Detection System (Applied Biosystems, Courtaboeuf, France). Cycling parameters were as follows: 95 ºC for 30 s; 40 cycles at 95 ºC for 5 s, 55 ºC for 30 s, and 72 ºC for 40 s; and one dissociation step of 95 ºC for 15 s, 60 ºC for 30 s, and 95 ºC for 15 s. All samples were analyzed in triplicate, and the 16S rRNA gene was used as an internal control housekeeping gene to normalize the levels of expression between samples. The real-time RT-PCR data were analyzed by the (ΔΔCt) method described in Applied Biosystems User Bulletin No. 2.

**Table 1:** Primers used for Real-time RT-PCR.

Primer	N315 ORF^*^	Sequence
*16S rRNA* sense	SArRNA01	5’-CGTGCTACAATGGACAATACAAA-3’
*16S rRNA *antisense	SArRNA01	5’-ATCTACGATTACTAGCGATTCCA-3’
*hla* sense	SA1007	5’-TGAATCCTGTCGCTAATG-3’
*hla* antisense	SA1007	5’-TATCATCCGACCTTTCACT-3’

^* ^ORF, open reading frame.

### 3.7. Statistical analysis of the results

Experimental data were analyzed using SPSS 12.0 statistical software. Data are expressed as the mean ± SD. Statistical differences were examined using the independent Student t-test. A *p* value less than 0.05 was considered statistically significant.

## 4. Conclusions

In the present study, we have investigated the influence of magnolol on the secretion of α-toxin by *S. aureus*. Our results showed that subinhibitory concentrations of magnolol dose-dependently inhibited the production of α-toxin in both MSSA and MRSA. These findings indicated that the structure of magnolol may be used as a basic structure for the development of drugs aimed at bacterial virulence factors.

## References

[1] Jabra-Rizk M.A., Meiller T.F., James C.E., Shirtliff M.E. (2006). Effect of farnesol on *Staphylococcus aureus* biofilm formation and antimicrobial susceptibility. Antimicrob. Agents Chemother..

[2] Koziel J., Maciag-Gudowska A., Mikolajczyk T., Bzowska M., Sturdevant D.E. (2009). Phagocytosis of *Staphylococcus aureus* by macrophages exerts cytoprotective effects manifested by the upregulation of antiapoptotic factors. PLoS One.

[3] Herbert S., Barry P., Novick R.P. (2001). Subinhibitory clindamycin differentially inhibits transcription of exoprotein genes in *Staphylococcus aureus*. Infect. Immun..

[4] Oscarsson J., Kanth A., Tegmark-Wisell K., Arvidson S. (2006). SarA is a repressor of *hla* (alpha-hemolysin) transcription in *Staphylococcus aureus*: its apparent role as an activator of *hla* in the prototype strain NCTC 8325 depends on reduced expression of *sarS*. J. Bacteriol..

[5] Peng H.L., Novick R.P., Kreiswirth B., Kornblum J., Schlievert P. (1988). Cloning, characterization, and sequencing of an accessory gene regulator (*agr*) in *Staphylococcus aureus*. J. Bacteriol..

[6] Giraudo A.T., Cheung A.L., Nagel R. (1997). The *sae* locus of *Staphylococcus aureus* controls exoprotein synthesis at the transcriptional level. Arch. Microbiol..

[7] Ho K.Y., Tsai C.C., Chen C.P., Huang J.S., Lin C.C. (2001). Antimicrobial activity of honokiol and magnolol isolated from *Magnolia officinalis*. Phytother. Res..

[8] Wang J.P., Ho T.F., Chang L.C., Chen C.C. (1995). Anti-inflammatory effect of magnolol, isolated from *Magnolia officinalis*, on A23187-induced pleurisy in mice. J. Pharm. Pharmacol..

[9] Wu S.N., Chen C.C., Li H.F., Lo Y.K., Chen S.A., Chiang H.T. (2002). Stimulation of the BK(Ca) channel in cultured smooth muscle cells of human trachea by magnolol. Thorax.

[10] Girgis D.O., Sloop G.D., Reed J.M., O'Callaghan R.J. (2005). Effects of toxin production in a murine model of *Staphylococcus aureus *keratitis. Invest. Ophthalmol. Vis. Sci..

[11] Tseng C.W., Stewart G.C. (2005). Rot repression of enterotoxin B expression in *Staphylococcus aureus*. J. Bacteriol..

[12] Bania J., Dabrowska A., Korzekwa K., Zarczynska A., Bystron J., Chrzanowska J., Molenda J. (2006). The profiles of enterotoxin genes in *Staphylococcus aureus* from nasal carriers. Lett. Appl. Microbiol..

[13] Trede N.S., Castigli E., Geha R.S., Chatila T. (1993). Microbial superantigens induce NF-kappa B in the human monocytic cell line THP-1. J. Immunol..

[14] Bernardo K., Pakulat N., Fleer S., Schnaith A., Utermöhlen O., Krut O., Müller S., Krönke M. (2004). Subinhibitory concentrations of linezolid reduce *Staphylococcus aureus* virulence factor expression. Antimicrob Agents Chemother..

[15] Koszczol C., Bernardo K., Krönke M., Krut O. (2006). Subinhibitory quinupristin/dalfopristin attenuates virulence of *Staphylococcus aureus*. J. Antimicrob. Chemother..

[16] Stevens D.L., Ma Y., Salmi D.B., McIndoo E., Wallace R.J. (2007). Impact of antibiotics on expression of virulence-associated exotoxin genes in methicillin-sensitive and methicillin-resistant *Staphylococcus aureus*. J. Infect Dis..

[17] Kuroda H., Kuroda M., Cui L., Hiramatsu K. (2007). Subinhibitory concentrations of beta-lactam induce haemolytic activity in *Staphylococcus aureus* through the SaeRS two-component system. FEMS Microbiol. Lett..

[18] Shah S., Stapleton P.D., Taylor P.W. (2008). The polyphenol (-)-epicatechin gallate disrupts the secretion of virulence-related proteins by *Staphylococcus aureus*. Lett. Appl. Microbiol..

[19] Qiu J., Jiang Y., Xia L., Xiang H., Feng H., Pu S., Huang N., Yu L., Deng X. (2010). Subinhibitory concentrations of licochalcone A decrease alpha-toxin production in both methicillin-sensitive and methicillin-resistant *Staphylococcus aureus* isolates. Lett. Appl. Microbiol..

[20] Smith-Palmer A., Stewart J., Fyfe L. (2004). Influence of subinhibitory concentrations of plant essential oils on the production of enterotoxins A and B and alpha-toxin by *Staphylococcus aureus*. J. Med. Microbiol..

[21] Cegelski L., Marshall G.R., Eldridge G.R., Hultgren S.J. (2008). The biology and future prospects of antivirulence therapies. Nat. Rev. Microbiol..

[22] Li M., Diep B.A., Villaruz A.E., Braughton K.R., Jiang X. (2009). Evolution of virulence in epidemic community-associated methicillin-resistant *Staphylococcus aureus*. Proc. Natl. Acad. Sci. USA.

[23] Gill S.R., Fouts D.E., Archer G.L., Mongodin E.F., Deboy R.T. (2005). Insights on evolution of virulence and resistance from the complete genome analysis of an early methicillin-resistant *Staphylococcus aureus* strain and a biofilm-producing methicillin-resistant *Staphylococcus epidermidis* strain. J. Bacteriol..

[24] Rowe G.E., Welch R.A. (1994). Assays of hemolytic toxins. Methods Enzymol..

[25] Laemmli U.K. (1970). Cleavage of structural proteins during the assembly of the head of bacteriophage T4. Nature (London).

[26] Sambanthamoorthy K., Smeltzer M.S., Elasri M.O. (2006). Identification and characterization of *msa* (SA1233), a gene involved in expression of SarA and several virulence factors in *Staphylococcus aureu*. Microbiology.

